# Local Catalytic Ignition during CO Oxidation on Low-Index Pt and Pd Surfaces: A Combined PEEM, MS, and DFT Study**

**DOI:** 10.1002/anie.201204031

**Published:** 2012-09-07

**Authors:** Diana Vogel, Christian Spiel, Yuri Suchorski, Adriana Trinchero, Robert Schlögl, Henrik Grönbeck, Günther Rupprechter

**Affiliations:** * Institute of Materials Chemistry, Vienna University of Technology, Getreidemarkt 9/BC/011060 Vienna (Austria); Department of Applied Physics and Competence Centre for Catalysis, Chalmers University of Technology41296 Göteborg (Sweden); Department of Inorganic Chemistry, Fritz-Haber-Institute of the Max-Planck-Society, Faradayweg 4–614195 Berlin (Germany)

**Keywords:** catalytic ignition, CO oxidation, DFT calculations, heterogeneous catalysis, photoemission electron microscopy

## Abstract

**Shedding light on light-off:**

: Photoemission electron microscopy, DFT, and microkinetic modeling were used to examine the local kinetics in the CO oxidation on individual grains of a polycrystalline sample. It is demonstrated that catalytic ignition (“light-off”) occurs easier on Pd(*hkl*) domains than on corresponding Pt(*hkl*) domains. The isothermal determination of kinetic transitions, commonly used in surface science, is fully consistent with the isobaric reactivity monitoring applied in technical catalysis.

Although modern catalytic converters significantly reduce pollution from automobile engines, a major amount of pollutants is still emitted just after “cold-start”, that is, until the catalytic converter warms up to its “ignition” temperature, at which the reaction rate rapidly switches from low to high conversion. There is thus substantial interest by manufacturers to reduce emissions during the start-up period of automotive catalysts. Consequently, sophisticated catalyst heating processes have been developed to quickly reach the critical temperature. Methods include operation at lean air-to-fuel ratio, exhaust-system combustion devices, secondary air injection into the exhaust, electrically heated catalysts, etc.^1^ Alternatively, as an “energy-neutral” approach, lowering the critical temperature seems more promising.

Catalytic ignition was originally considered as being solely a heat balance problem,^2^ with the critical ignition temperature being defined as the temperature when the heat generated by the exothermic reaction exceeds the dissipated heat (i.e. the catalyst heats up and no external heating is required anymore; this is termed “light-off”). In fact, the catalytic ignition process represents a convolution of reaction kinetics and heat generation, since the heat produced is governed by the reaction rate which in turn is determined by the reaction kinetics.^3^, ^4^

However, for CO oxidation under (ultra)high-vacuum, on model catalysts, the heat generation by the reaction can be neglected, and the catalytic ignition problem can thus be reduced to the “pure kinetics”, that is, to the temperature-triggered kinetic transition from the low-rate steady state regime to a high-rate steady state.^5^, ^6^ Such a transition in the CO oxidation reaction is a phenomenon of purely kinetic origin^7^, ^8^ which is well studied, mainly by varying the CO/O_2_ ratio.^9^–^12^

Herein we present an experimental and theoretical study of catalytic ignition in the CO oxidation reaction under high-vacuum conditions (10^−5^ mbar range), for μm-sized (*hkl*) domains of polycrystalline Pt and Pd foil. Emphasis is on local kinetic measurements enabling a direct comparison of the reaction properties of low-Miller-index Pt(*hkl*) and Pd(*hkl*) domains. To our knowledge this is the first time that the different metals and different terminations have been evaluated under basically identical reaction conditions, yielding the inherent reaction behavior. It is demonstrated that the isothermal and the isobaric determination of reactivity states yield equivalent results in the pressure regime considered in the present study on Pt and Pd foil. Regarding the reactivity range in (*p*,*T*) parameter space, Pd foil is a better-suited catalyst for CO oxidation than Pt foil because Pd is poisoned and also reactivated at higher CO-to-oxygen pressure ratios (i.e. Pd is more CO tolerant). These experimental findings are supported and explained by density functional theory (DFT) calculations.

Recently, we have developed an experimental approach, based on the analysis of local PEEM (photoemission electron microscopy) intensities, which allows the in situ observation of kinetic phase transitions on individual differently oriented grains of a polycrystalline Pt foil.^12^ The idea of the experiment is based on the fact that the local photoemission yield is directly dependent on the local CO or oxygen coverage (through the local work function). Since the CO or oxygen coverage governs the rate of CO_2_ formation,^13^ the local PEEM image intensity serves as an indicator for the local reaction rate, which allows “imaging” of the kinetic phase transitions on the μm-scale. Previously, we have used such an analysis to construct kinetic phase diagrams for the CO oxidation reaction in the 10^−5^ mbar pressure range for individual [100]-, [110]-, and [111]-oriented grains of a Pt foil.^12^ The single (*hkl*) domains exhibited independent reaction properties, analogous to the corresponding single crystals, and the sum of the local kinetic diagrams for individual grains is equal to the global kinetic diagram obtained for the whole sample by mass spectrometry (MS; indicating that the grain boundaries do not contribute directly to the overall reactivity and that all relevant catalytic processes were monitored by this approach).^12^ Herein, this approach has been extended to Pd samples and to the reaction light-off on Pt and Pd foil at isobaric conditions.

A series of ignition/extinction experiments has been performed in a UHV chamber with a base pressure below 10^−9^ mbar on polycrystalline Pd (AlfaAesar, 99.9 %) and Pt (MaTecK, 99.99 %) foil, both consisting of up to 100 μm large crystallites of different surface orientation, as determined by work-function analysis.^12^ The reaction was followed in situ (Figure 1a), simultaneously by PEEM and by quadrupole mass spectrometry (QMS). QMS provides the average CO_2_ formation rate originating from all the grains of the polycrystalline foil (Figure 1b), whereas the recorded PEEM sequences deliver the local photoemission yield from individual grains (Figure 1c). The PEEM data provides laterally resolved kinetic information from the different domains at strictly identical experimental conditions, since all domains of the foil are exposed to the same gas-phase composition at the same temperature. In this way, the global (Figure 1b) and local (Figure 1c) kinetics for the Pd foil can be compared, for example, for a typical temperature scan from 372 K to 493 K, with a rate of 0.5 K s^−1^ at constant *p*_CO_=5.8×10^−6^ mbar and ρ _o_2__=1.3×10^−5^ mbar. Following the temperature ramp, the global CO_2_ rate suddenly increases indicating the transition *τ*_B_* from the state of low catalytic activity (CO poisoned surface; video frame 1 in Figure 1b, dark contrast) to the state of high catalytic activity at which the surface becomes oxygen covered (frame 4; bright contrast). Analogous to the MS signal in the overall CO_2_ reaction rate, the jumps in the local PEEM intensity represent the local kinetic transitions on the individual grains (Figure 1c). These transitions do not occur simultaneously on the different orientations but show a pronounced structure sensitivity with clearly identifiable critical temperatures of 417 K for Pd(110), 423 K for Pd(100), and 432 K for Pd(111). A similar observation was made for the reaction extinction, that is, for the transition *τ*_A_* from the high-reactivity to the low-reactivity state upon cooling the sample. Again, the curve of the *global* CO_2_ production rate appears to be “smoothened out” (black curve in Figure 1 b), whereas *local* extinction on the individual grains occurs rather sharply and independently from each other (Figure 1 c). This clearly shows the limitation of averaging techniques, such as mass-spectroscopy, which can not reveal the important local kinetics.

**Figure 1:**
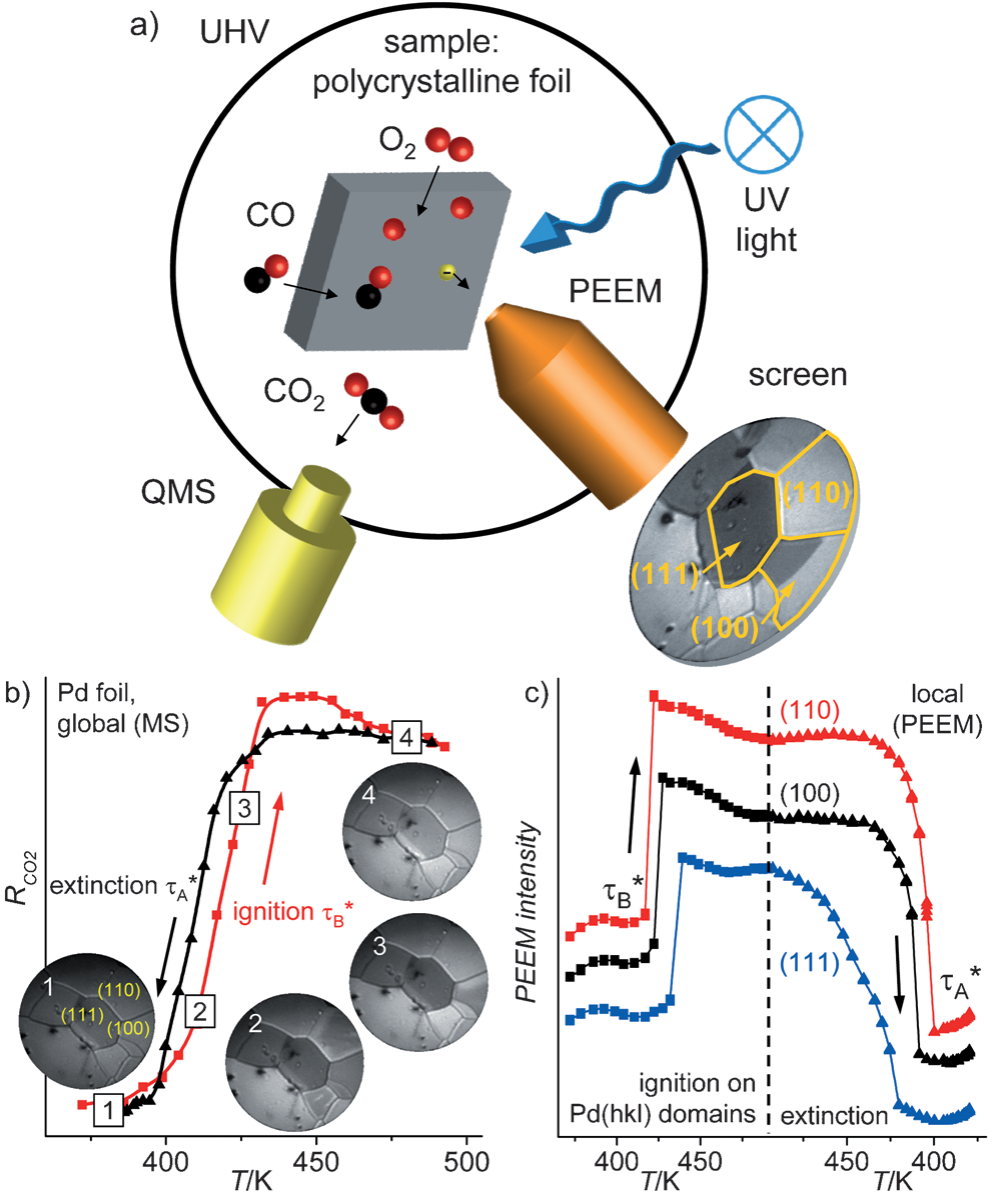
a) Scheme of the experiment: The CO oxidation reaction on polycrystalline Pd (Pt) foil is simultaneously monitored by MS and PEEM. Three different domains, Pd(110), Pd(100), and Pd(111) are identified in the PEEM image. b) Ignition (red squares) and extinction curves (black triangles) on Pd foil, as CO_2_ production rate measured globally by MS during cyclic variation of the sample temperature (rate: 0.5 K s^−1^) at constant ρ_CO_=5.8×10^−6^ mbar and ρ_o_2__ = 1.3×10^−5^ mbar. Simultaneously recorded PEEM video-sequences illustrate the ignition process: Frame (1) inactive, CO covered surface; Frame (2)—ignition begins on (110) domains; Frame (3)—ignition continues on (100) domains; Frame (4)—oxygen covered, active surface. c) Laterally resolved ignition/extinction measurements: local PEEM intensity for the individual (110), (100), and (111) domains during the same cyclic temperature scan as in (b). The vertical dashed line indicates the turning point from heating to cooling.

Usually, kinetic transitions in CO oxidation were experimentally studied under high-vacuum conditions by varying the CO/O_2_ pressure ratio at constant temperature.^9^, ^10^, ^12^ Such an experiment is illustrated for Pd foil in the right inset of Figure 2a, at constant ρ _o_2__=1.3×10^−5^ mbar and *T*=449 K. Similar to the case of Pt foil,^12^, ^14^, ^15^ the global CO_2_ formation rate exhibits a pronounced hysteresis upon cyclic variation of the CO partial pressure manifest by the gap between the kinetic transition *τ*_A_ from the high-reactivity to the low-reactivity state and the reverse transition *τ*_B_. In between, the system is bistable, that is, it can be either in the high- or in the low-reactivity steady state, depending on the prehistory. The *τ*_A_ and *τ*_B_ points are temperature-dependent, thus a (global) kinetic phase diagram can be constructed for the Pd foil, summarizing the kinetic transitions (Figure 2a).

**Figure 2:**
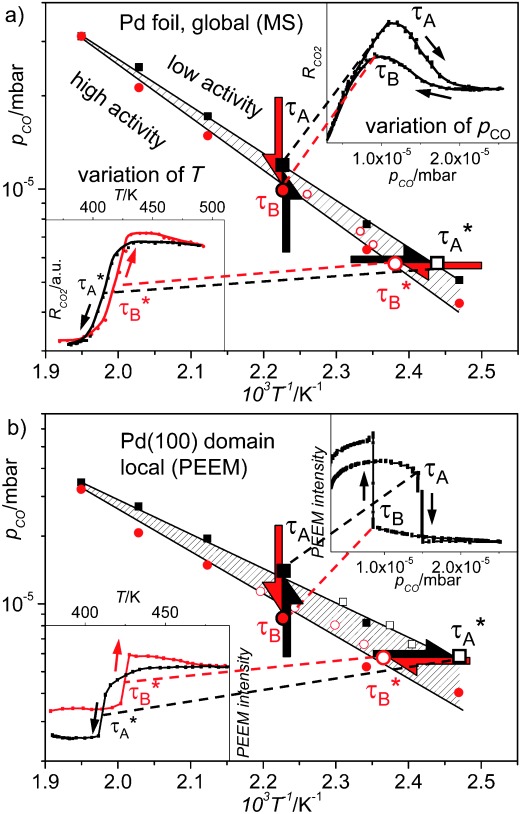
Global (a) and local (b) kinetic phase diagram illustrating the CO oxidation reaction on polycrystalline Pd foil (a) and on a single Pd(100) domain of the Pd foil (b). Note the agreement of the transition points *τ*_A_* and *τ*_B_* obtained at varying *T* (from the ignition/extinction curves shown in the left insets; open symbols) with the diagram obtained from cyclic variation of *p*_CO_ (from the poisoning/reactivation curves in the right insets; filled symbols). The dashed regions indicate the range of bistability.

For comparison, the kinetic transition points (*τ*_A_*/*τ*_B_*) extracted from isobaric ignition/extinction experiments (shown in the left inset of Figure 2a for constant *p*_CO_=5.8×10^−6^ mbar and ρ_o_2__=1.3×10^−5^ mbar) are also plotted into the isothermally obtained diagram in Figure 2a. The *isobaric* variation of the reaction temperature results in kinetic transitions which quantitatively match the kinetic phase diagram obtained by the *isothermal* variation of the CO pressure. In Figure 2b the result of the local PEEM analysis of the same transition for an individual Pd(100) domain is shown: again, the left inset shows the ignition/extinction experiment and the right inset presents the transitions obtained by variation of the CO pressure at constant *T* and *p*_o_2__. As in the case of the (global) MS data, the transition points obtained from isobaric experiments are in quantitative agreement with the isothermal experiments, thus linking the typical surface science (isothermal) approach and the typical technical catalysis (isobaric) approach.

Figure 3a, allows the *global* reaction behavior of Pt and Pd foil to be compared, whereas in Figure 3b the *local* kinetic transitions of the individual Pt(*hkl*) domains are contrasted with those of the Pd(*hkl*) domains. The most striking differences between Pt and Pd are: 1) the global and the respective local kinetic phase diagrams of Pd foil are situated at significantly higher CO partial pressure and 2) the bistability range is much narrower for Pd than for Pt foil. In particular, this means that for Pd, the transition *τ*_A_ from the high to the low reactivity state occurs at higher CO partial pressure than for Pt, and that the reverse transition *τ*_B_ also occurs at a higher CO-to-oxygen ratio than for Pt. In other words, Pd is the better (more CO-tolerant) catalyst than Pt under the current conditions because *more* CO is needed to poison the Pd surface and a *lower* oxygen-to-CO ratio is sufficient to “reactivate” the Pd surface. In addition, the bistability regime of Pd disappears at a lower temperature than that of Pt, namely at *T*_Pd_=513 K in contrast to *T*_Pt_=573 K, so even at lower temperature Pd cannot be poisoned by CO anymore.

**Figure 3:**
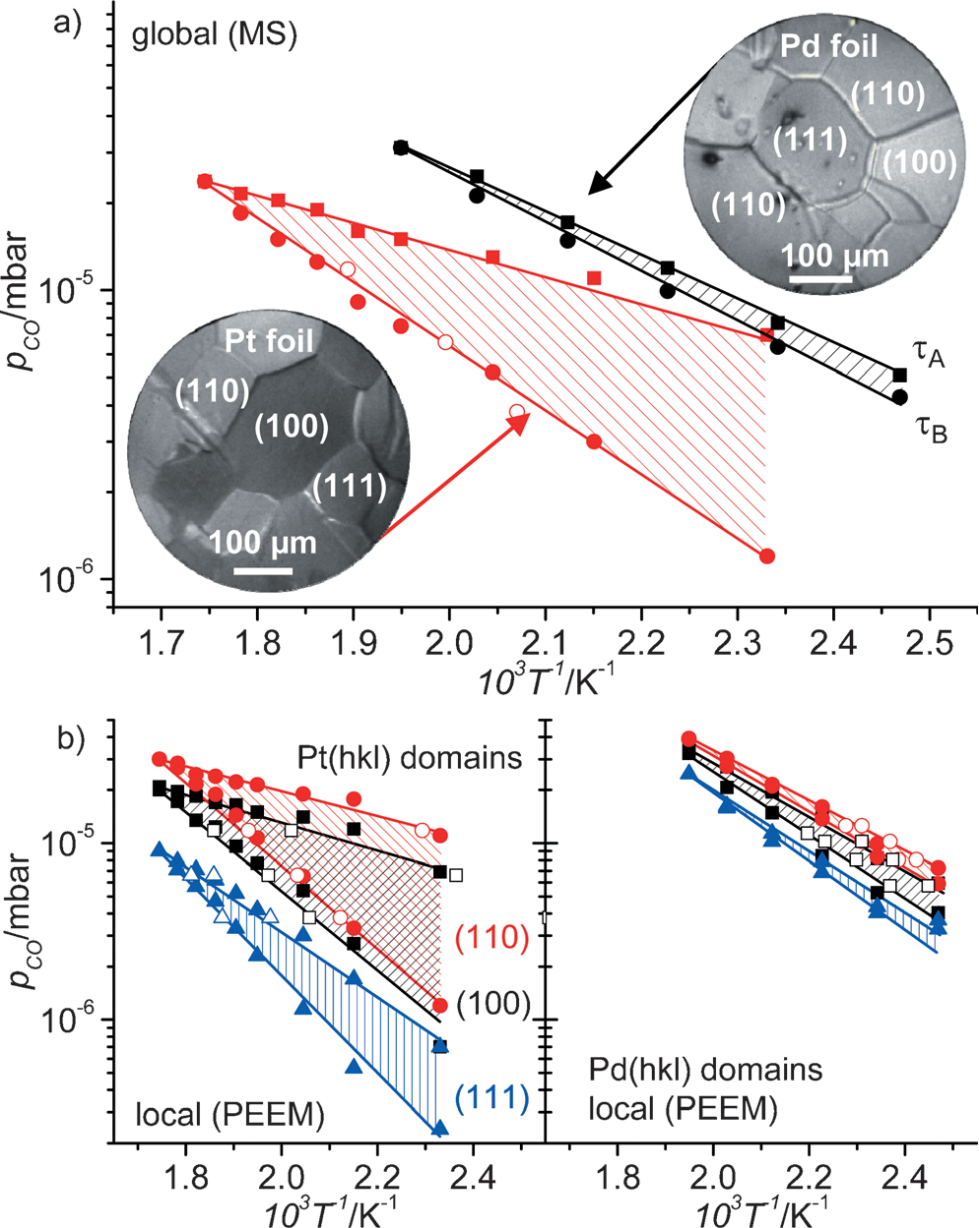
Palladium versus platinum in CO oxidation. a) Comparison of the global kinetic phase diagrams (by MS) at constant oxygen pressure (ρ_o_2__=1.3×10^−5^ mbar) of polycrystalline Pt (filled red squares and circles) and Pd (black squares and circles). (An improved temperature measurement method was used in comparison to Ref. 12, with the corresponding correction being applied to the Pt data.) Open circles are ignition points for Pt. b) Corresponding local kinetic phase diagrams for individual Pt(*hkl*) domains (left) and Pd (right), obtained by local PEEM intensity analysis. Open symbols are the local ignition and extinction points.

To explain these observations, density functional theory (DFT) has been applied to calculate the adsorption energies of CO and oxygen on clean Pd(*hkl*) and Pt(*hkl*) domains, as well as the reaction barriers (cf. Table S1 in the Supporting Information). Literature values for the sticking coefficients of CO and oxygen and the desorption temperatures of CO on Pd(*hkl*) and Pt(*hkl*), are also included in Table S1. The bistability regions for Pt(111), Pt(100), Pt(110), and Pd(111) have been simulated by a micro-kinetic model (for details see Supporting Information) based on the conventional Langmuir–Hinshelwood mechanism. In Figure 4a the calculated bistability regions are compared for Pd(111) and Pt(111), and in Figure 4b the three low Miller-indexed surfaces of Pt can be compared to each other. The right inset in Figure 4a depicts a simulated ignition/extinction curve for the Pd(111) surface, and the left inset shows a simulated hysteresis curve for the Pt(111) surface resulting from *p*_CO_ variation. In all cases, the kinetic simulations are in agreement with the experiment: the bistability region of Pd is considerably narrower than in case of Pt, and the kinetic phase diagram of Pd is located at higher CO pressure. The DFT-derived order of the local kinetic phase diagrams of the three Pt surfaces also corresponds to the experimental results: The Pt(100) surface is active at higher CO pressures than Pt(111), and Pt(110) is active at the highest CO pressures.

**Figure 4:**
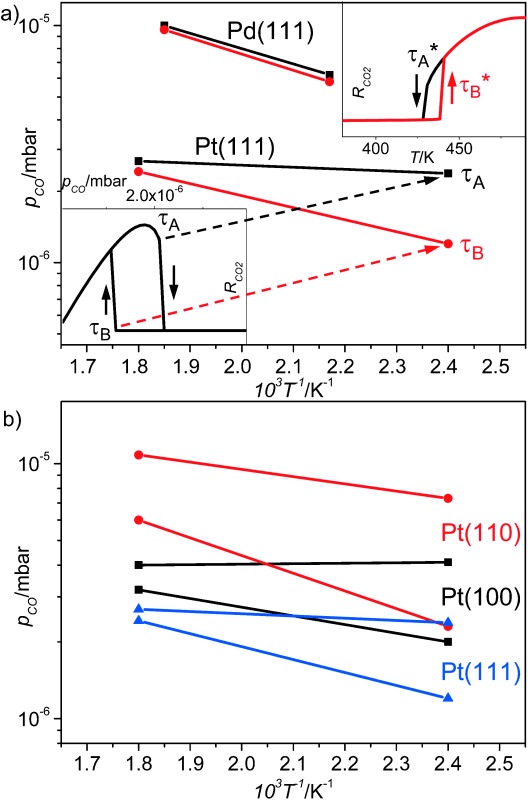
a) Simulated kinetic phase diagrams for Pd(111) and Pt(111) at ρ_o_2__=1.3×10^−5^ mbar, as well as a simulated *p*_CO_ hysteresis curve for Pt(111) at 417 K (left inset) and a simulated ignition/extinction curve for Pd(111) at *p*_CO_=5.8×10^−6^ mbar (right inset). b) Simulated local kinetic phase diagrams of Pt(110), Pt(100), and Pt(111) at ρ_o_2__=1.3×10^−5^ mbar.

The DFT calculations and the kinetic modeling provide an unambiguous way to rationalize the experimental findings. The experiments showed that at a given oxygen pressure and temperature the Pt(*hkl*) domains are deactivated at lower CO pressure than Pd(*hkl*). This can mainly be attributed to the higher adsorption energies of oxygen on Pd(*hkl*) than on Pt(*hkl*) (cf. Table S1, Supporting Information), that is, oxygen is more strongly bound to Pd and thus, the CO-poisoning of the surface occurs at higher CO pressures. Regarding the transition *τ*_B_ from the low- to the high-reactivity state, the Pd foil “reactivates” at a considerably higher CO pressure than Pt foil. This results from the higher sticking coefficients of oxygen for the Pd(*hkl*) domains compared to the Pt(*hkl*) domains: whereas CO adsorption properties are quite similar on Pt and Pd(*hkl*) surfaces, oxygen adsorption is clearly favored on Pd(*hkl*). The disappearance of the bistability regime at the so-called “cusp-point” occurs at lower temperature for Pd than for Pt foil. This result can be explained by the generally lower desorption temperature of CO on Pd(*hkl*).

In summary, local domain-specific kinetic measurements for individual Pd and Pt crystalline grains have been performed under identical reaction conditions, allowing a direct comparison of inherent catalytic properties of Pt(*hkl*)- and Pd(*hkl*)-domains with respect to CO oxidation. It has been shown that for the CO oxidation reaction the typical surface-science approach for determining reaction kinetics, that is, the isothermal monitoring of kinetic transitions, quantitatively yields the same results as the common technical catalysis approach, that is, the isobaric study of the different reactivity regimes, at least in the pressure range of around 10^−5^ mbar. The differences observed in the catalytic activity of Pt(*hkl*) and Pd(*hkl*) domains, such as the much higher CO-tolerance and the higher reactivation ability of Pd compared to Pt, as well as the differences between particular crystallographic orientations, were rationalized by DFT calculations and kinetic reaction modeling.
