# Supplementary material for: Local Catalytic Ignition during CO Oxidation on Low-Index Pt and Pd Surfaces: A Combined PEEM, MS, and DFT Study
Source: Angew Chem Int Ed Engl. 2012 Sep 7;51(40):10041–4. doi: 10.1002/anie.201204031 (PMC3482928; doi:10.1002/anie.201204031)
Supplement: Supplementary file 1 [file anie0051-10041-SD1.pdf]

Angewandte Chemie
Eine Zeitschrift der Gesellschaft Deutscher Chemiker

Supporting Information

© Wiley-VCH 2012

69451 Weinheim, Germany

Local Catalytic Ignition during CO Oxidation on Low-Index Pt and Pd Surfaces: A Combined PEEM, MS, and DFT Study**

*Diana Vogel, Christian Spiel, Yuri Suchorski, Adriana Trinchero, Robert Schlögl, Henrik Grönbeck, and Günther Rupprechter**

anie_201204031_sm_miscellaneous_information.pdf

Supporting Information

S1. Global ignition/extinction measurements for Pt foil were performed by mass spectrometric monitoring of the CO_2 reaction rate at constant p_{O2} = 1.3x10^{-5} mbar and p_{CO} = 6.6 x10^{-6} mbar, as illustrated in Figure S1a. The global extinction curve appears to be smoothened out and a distinct “global extinction temperature” can hardly be assigned. This “smoothing” effect is caused by sequential extinction of differently oriented Pt(hkl) domains, i.e. at isobaric conditions the local extinctions on the individual Pt(hkl) domains occur sequentially within a broad temperature range which covers almost the whole global hysteresis loop. For comparison, a global R_{CO2} hysteresis curve obtained under isothermal conditions upon cyclic variation of the CO partial pressure is shown in Figure S1b. The smooth appearance of the transition τ_A upon CO pressure variation reflects again the sequential CO-poisoning of the (hkl) domains.

Parallel to the global MS measurements shown in Figures S1a and b, the local PEEM intensities from individual grains of the Pt foil were monitored, as shown in Figure S1c and d for a (100) domain. For both type of measurements, variation of temperature (Figure S1c) and variation of CO partial pressure (Figure S1d), the local kinetic transition points τ_A*/τ_B* and τ_A/τ_B, respectively, are much more pronounced than in the global MS measurements (Figures S1a, b).

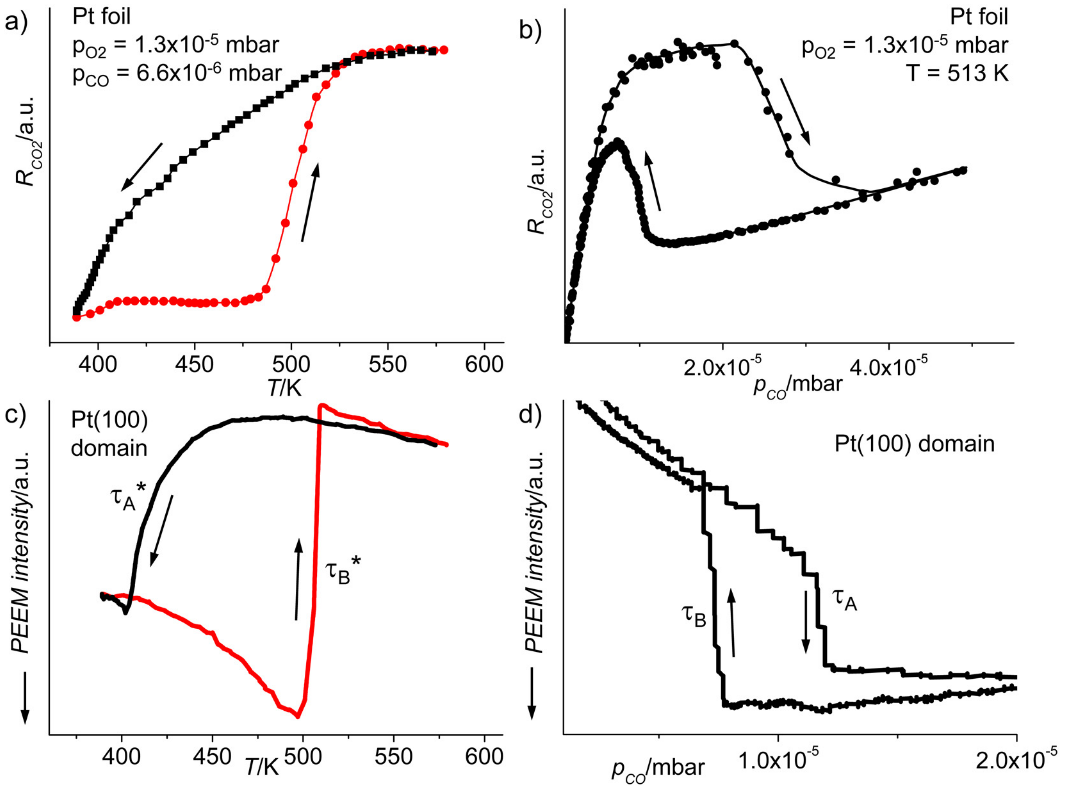

Figure S1. a) Global ignition (red) and extinction (black) R_{CO2} curves obtained for Pt foil upon cyclic temperature variation at constant p_{CO} = 6.6x10^{-6} mbar and p_{O2} = 1.3x10^{-6} mbar; b) global hysteresis in the CO_2 rate upon p_{CO} variation at T = 513 K, at the same oxygen pressure as in a); c) local PEEM intensity during ignition/extinction on a Pt(100) domain, simultaneously obtained with the R_{CO2} curve in a); d) local PEEM intensity hysteresis on a Pt(100) domain during CO pressure variation as shown in b).

S2. To obtain local ignition/extinction of Pt(hkl) domains, PEEM video-sequences were recorded simultaneously to the MS-monitoring, under isobaric reaction conditions (p_{CO} = 6.6x10^{-6} mbar and p_{O2} = 1.3x10^{-5} mbar). Details of the identification of the surface orientation of the individual Pt(hkl) domains and the contrast mechanism (CO-covered surface appears bright in

comparison to oxygen covered surface) were described in Ref. [S1]. Note that on Pt, contrary to Pd, the oxygen covered surface appears darker in PEEM than CO covered regions.

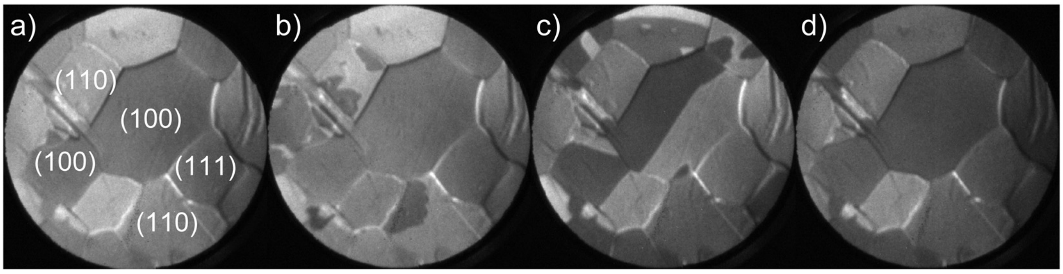

Figure S2. Sequence of PEEM images during the ignition τ_B* on Pt foil at constant p_{CO} = 6.6x10^{-6} mbar and p_{O2} = 1.3x10^{-5} mbar. The temperature is ramped with a heating rate of ~0.5 K/s from 483 K in frame (a) (CO-covered), to 492 K and 506 K in frame (b) and (c) (ignition on (110) and (100) domains) and to 568 K in frame (d) (oxygen covered). The orientation of the individual domains is indicated in frame a).

S3. Density Functional Theory Calculations

Density functional theory (DFT) was used in a real space grid implementation[S2, S3] of the projector augmented wave (PAW) method[S4] with a grid spacing of 0.18 Å. The frozen core and projectors were generated with scalar relativistic corrections for Pd and Pt. Exchange and correlation contributions were described by the spin-polarized Perdew-Burke-Ernzerhof (PBE) functional.[S5] Reciprocal space integration over the Brillouin zone was approximated with finite sampling.[S6, S7] An effective temperature of 0.1 eV was used to smear the Fermi discontinuity. Activation energies were evaluated with the nudged-elastic band method[S8] or constrained optimization.

Three surfaces were considered for Pd and Pt, namely (111), (100) and the missing row reconstructed (110). Four atomic layers were used to model the slabs. The calculations were performed with an adsorption coverage of 0.25. The structural relaxations were performed within the quasi-Newton method. Structures are regarded optimized when the largest element of the gradient was smaller than 0.05 eV/Å.

The energetic results from the DFT are collected in Table S1. Only adsorption of atomic oxygen was considered. Thus, the $E_{ads}(O_2)$ value in Table S1 corresponds to the adsorption energy of two oxygen atoms with respect to O_2 in the gas phase. The results are in good agreement with previous reports. It should be noted that the reaction barrier is sensitive to the assumed reaction path. For the (111) surfaces, the barrier is obtained from CO close to an atop position and O in a bridge configuration. CO_2 is in this case formed over an fcc hollow site. Over the (100) surfaces, CO_2 is formed from both CO and O in opposite bridge positions. On (110), the barrier is evaluated with CO in a bridge position on the row and O in a hollow position.

Table S1: Adsorption energies of CO and O_2 and the barrier (E_r) to form CO_2 from adsorbed CO and O on the three low Miller index planes of Pt and Pd, as well as the sticking coefficients of CO and oxygen and the desorption temperature of CO, as known from literature. The energies are reported in eV. The sticking coefficients used in the simulations are given in parentheses. The preferred adsorption sites are indicated for CO and O; atop (t), bridge (b), hollow (h) and fcc hollow (fcc).

	**E_{ads}(CO)**	**$E_{ads}(O_2)$**	**E_r**	**$s_0(O_2)$**	**s_0(CO)**	**T_{des}(CO) [K]**
**Pt(110)**	2.00 (t)	2.40 (h)	0.96	0.3-0.4[S9] (0.4)	0.8-1[S10] (0.9)	500-520[S10]
**Pt(100)**	2.02 (b)	2.14 (b)	0.55	10^{-3}/0.1[S11] (0.1)	0.6-0.75[S12] (0.6)	~520[S13]
**Pt(111)**	1.76 (fcc)	2.28 (fcc)	0.95	0.06[S14] (0.1)	0.6-0.8[S15] (0.9)	400-450[S16]
**Pd(110)**	1.97 (h)	2.48 (h)	-	0.96[S17]	1[S18]	400/475[S19]
**Pd(100)**	1.84 (b)	2.40 (h)	0.80	0.1-0.75[S20, S21]	0.6-0.8[S22]	~480[S22]
**Pd(111)**	1.96 (fcc)	2.50 (fcc)	1.53	0.7[S23] (0.7)	0.96[S24] (0.7)	450[S25]

S4. Micro-Kinetic Model

The micro-kinetic model is based on the conventional Langmuir-Hinshelwood mechanism for CO oxidation:

$CO + * \leftrightarrow CO*$ (R1)

$O_2 + 2* \leftrightarrow 2O*$ (R2)

$O* + CO* \leftrightarrow CO_2 + 2*$ (R3)

Here, * denotes a free surface site and X* an adsorbate X bonded to a surface site. The corresponding rate equations are:

$$\frac{d\theta_{CO}}{dt} = s_{CO}^0(1-\theta_{CO}^2)p_{CO}k_{CO}^a - k_{CO}^d\theta_{CO} - k^r\theta_{CO}\theta_O$$

$$\frac{d\theta_O}{dt} = 2s_O^0(1-\theta_{CO}-\theta_O)^2 p_{O_2}k_{O_2}^a - k_{O_2}^d\theta_O^2 - k^r\theta_{CO}\theta_O$$

Here, θ_{CO} and θ_O are the coverages, p_{CO} and p_{O_2} are the pressures, and s_{CO}^0 and $s_{O_2}^0$ the initial sticking coefficients. The rate constants are given by:

$$k_{CO}^d = \nu_{CO}^d e^{-E_{CO}^d(1-\alpha\theta_{CO})/k_BT}$$

$$k_{O_2}^d = \nu_{O_2}^d e^{-E_{O_2}^d(1-\alpha\theta_O)/k_BT}$$

$$k^r = \nu^r e^{-E^r(1-\beta\theta_{CO})/k_BT}$$

$$k_X^a = 1/N_0\sqrt{2\pi Mk_BT}$$

T is the temperature, k_B is the Boltzmann constant, M is the mass of the molecule under consideration, and N_0 is the number of sites per area. A linear coverage dependence is introduced for the adsorption energies and the barrier. α and β are in all cases set close to 0.5. The coverage dependence (strength and functional form) could, in principle, be evaluated from first principles. However, here we have followed the strategy in Ref. [S26]. The desorption of O_2 is included above for completeness. The kinetics is insensitive to this step and it was not included in the simulations. The pre-exponential factors are set to $\nu_{CO}^d = 10^{15}$ and $\nu^r = 10^{14}$, respectively [S26].

References

[S1] Y. Suchorski, C. Spiel, D. Vogel, W. Drachsel, R. Schlögl, G. Rupprechter, *ChemPhysChem* **2010**, *11*, 3231-3235.

[S2] J. J. Mortensen, L. B. Hansen, K. W. Jacobsen, *Phys. Rev. B* **2005**, *71*, 035109.

[S3] https://wiki.fysik.dtu.dk/gpaw.

[S4] P. E. Blöchl *Phys. Rev. B* **1994**, *50*, 17953-17979.

[S5] J. P. Perdew, K. Burke, M. Ernzerhof, *Phys. Rev. Lett.* **1996**, *77*, 3865-3868.

[S6] H. J. Monkhorst, J. D. Pack, *Phys. Rev. B* **1976**, *13*, 5188-5192.

[S7] J. D. Pack, H. J. Monkhorst, *Phys. Rev. B* **1977**, *16*, 1748-1749.

[S8] G. Henkelman, B. P. Uberuaga, H. J. Jónsson, *J. Chem. Phys.* **2000**, *113*, 9901-9904.

[S9] R. Ducros, R. Merrill, *Surf. Sci.* **1976**, *55*, 227-245.

[S10] R. McCabe, L. Schmidt, *Surf. Sci.* **1976**, *60*, 85-98.

[S11] P. Norton, K. Griffiths, P. Bindner, *Surf. Sci.* **1984**, 138, 125-147.

[S12] G. Brodén, G. Pirug, H. Bonzel, *Surf. Sci.* **1978**, *72*, 45-52.

[S13] R. J. Behm, P. A. Thiel, P. R. Norton, G. Ertl, *J. Chem. Phys.* **1983**, *78*, 7437-7447.
[S14] Y. Y. Yeo, L. Vattuone, D. A. King, *J. Chem. Phys.* **1997**, *106*, 392-401.
[S15] C. T. Campbell, G. Ertl, H. Kuipers, J. Segner, *Surf. Sci.* **1981**, *107,* 207-219.
[S16] G. Ertl, M. Neumann, K. M. Streit, *Surf. Sci.* **1977***, 64*, 393-410.
[S17] K. Yagi, D. Sekiba, H. Fukutani, *Surf. Sci.* **1999***, 442*, 307-317.
[S18] J. Goschnick, M. Grunze, J. Loboda-Cackovic, J. H. Block, *Surf. Sci.* **1987**, *189-190*, 137-146.
[S19] J. Goschnick, M. Wolf, M. Grunze, W. N. Unertl, J. H. Block, J. Loboda-Cackovic, *Surf. Sci.* **1986**, *178*, 831-841.
[S20] T. Orent, S. Bader, *Surf. Sci.* **1982***, 115*, 323-334.
[S21] A. Gerbi, L. Savio, L. Vattuone, F. Pirani, D. Cappelletti, M. Rocca, *Angew. Chem.* **2006**, *118*, 6807-6810; *Angew. Chem. Int. Ed.* **2006**, *45*, 6655-6658.
[S22] R. J. Behm, K. Christmann, G. Ertl, M. A. van Hove, *J. Chem. Phys.* **1980**, *73*, 2984-2995.
[S23] F. P. Leisenberger, G. Koller, M. Sock, S. Surnev, M. G. Ramsey, F. P. Netzer, B. Klötzer, K. Hayek, *Surf. Sci.* **2000***, 445*, 380-393.
[S24] T. Engel, *J. Chem. Phys.* **1978***, 69*, 373-385.
[S25] X. Guo, J. T. Yates, *J. Chem. Phys.* **1989**, *90*, 6761-6766.
[S26] P.-A. Carlsson, M. Skoglundh, P. Thormählen and B. Andersson, *Top. Catal.* 2004, *30/31*, 375-381.
